# Heterogeneity of quality of life in young people attending primary mental health services

**DOI:** 10.1017/S2045796022000427

**Published:** 2022-07-20

**Authors:** Sue M. Cotton, Matthew P. Hamilton, Kate Filia, Jana M. Menssink, Lidia Engel, Cathrine Mihalopoulos, Debra Rickwood, Sarah E. Hetrick, Alexandra G. Parker, Helen Herrman, Nic Telford, Ian Hickie, Patrick D. McGorry, Caroline X. Gao

**Affiliations:** 1 Centre for Youth Mental Health, The University of Melbourne, Parkville, VIC 3052, Australia; 2 Orygen, Parkville, VIC 3052, Australia; 3 Deakin Health Economics, Faculty of Health, Institute for Health Transformation, School of Health and Social Development, Deakin University, Geelong, Australia; 4 headspace National Youth Mental Health Foundation Ltd, Melbourne, VIC 3000, Australia; 5 Faculty of Health, University of Canberra, Canberra, ACT 2617, Australia; 6 Department of Psychological Medicine, The University of Auckland, Auckland 1142, New Zealand; 7 Victoria University, Institute for Health and Sport, Melbourne, VIC 8001, Australia; 8 Brain and Mind, University of Sydney, Camperdown, NSW 2050, Australia; 9 Department of Epidemiology and Preventive Medicine, Monash University, Melbourne, VIC 3004, Australia

**Keywords:** Adolescence, latent class analysis, mental health services, quality of life

## Abstract

**Aims:**

The utility of quality of life (QoL) as an outcome measure in youth-specific primary mental health care settings has yet to be determined. We aimed to determine: (i) whether heterogeneity on individual items of a QoL measure could be used to identify distinct groups of help-seeking young people; and (ii) the validity of these groups based on having clinically meaningful differences in demographic and clinical characteristics.

**Methods:**

Young people, at their first presentation to one of five primary mental health services, completed a range of questionnaires, including the Assessment of Quality of Life–6 dimensions adolescent version (AQoL-6D). Latent class analysis (LCA) and multivariate multinomial logistic regression were used to define classes based on AQoL-6D and determine demographic and clinical characteristics associated with class membership.

**Results:**

1107 young people (12–25 years) participated. Four groups were identified: (i) no-to-mild impairment in QoL; (ii) moderate impairment across dimensions but especially mental health and coping; (iii) moderate impairment across dimensions but especially on the pain dimension; and (iv) poor QoL across all dimensions along with a greater likelihood of complex and severe clinical presentations. Differences between groups were observed with respect to demographic and clinical features.

**Conclusions:**

Adding multi-attribute utility instruments such as the AQoL-6D to routine data collection in mental health services might generate insights into the care needs of young people beyond reducing psychological distress and promoting symptom recovery. In young people with impairments across all QoL dimensions, the need for a holistic and personalised approach to treatment and recovery is heightened.

## Introduction

Quality of life (QoL) has become an important health outcome measurement concept for understanding the effectiveness of treatment, evaluating service provision and informing resource allocation (Torrance, 1987; Aaronson, 1988; Higginson and Carr, 2001). The World Health Organization (WHO) defines QoL as ‘individuals' perception of their position in life in the context of the culture and value systems in which they live and in relation to their goals, expectations, standards and concerns’ (Whoqol Group, 1995). Because of the subjective nature of QoL, it is generally assessed using self-report measures. Examples of well-known QoL measures include the World Health Organisation Quality of Life Scale (WHOQoL; Whoqol Group, 1995), the Medical Outcomes Study 36-item Short Form Survey (SF-36; Ware *et al*., 1996), the EuroQoL (EQ-5D; EuroQol group, 1990) and the Assessment of Quality of Life (AQoL; Hawthorne *et al*., 1999). Unlike disease-specific scales, these generic QoL measures have the advantage of allowing comparisons between individuals with different health conditions.

As a framework that reflects individuals' satisfactions and preferences, QoL is well aligned with the holistic, client-centred principles that have guided international efforts to reform mental health care for young people. We know that young people with mental ill-health, especially internalising disorders such as mood disorders, have lower QoL than their peers (Weitkamp *et al*., 2013) and those with physical disorders (Sawyer *et al*., 2002). We also know that there is much heterogeneity in presentation, illness course, degree of complexity and outcome of youth mental illness (Hansell *et al*., 2012; Scott *et al*., 2013, 2018; Hickie *et al*., 2019). Symptom measures alone (e.g., measures of psychological distress) are non-specific and may not provide the clinician with meaningful information on how to improve holistic care for the young person. Subjective perception of health status across multiple domains as assessed by QoL measures, can be used to optimise an individual's treatment (Ravens-Sieberer *et al*., 2014). Clinicians can then work to address domains of concern for the individual, providing more targeted or personalised approaches to care (e.g., improving housing where the person has indicated dissatisfaction). However, the extent to which QoL data can be used to better understand complexity in youth mental illness and tailor appropriate treatments and allocation of clinical resources is underexplored.

QoL has long been recognised as a multidimensional construct (Aaronson, 1988), falling into four major categories of physical, social, functional and mental health (Aaronson, 1988; Bullinger and Quitmann, 2014). However, in most contexts, a single number has been used to denote a person's QoL on the questionnaire. This could be a simple addition of a person's answers on the scale (e.g., an overall global score) or for some generic QoL measures, a preference-weighted utility score. The problem with single number scores is that meaningful information pertaining to individuals' QoL is lost and inter-individual variability is ignored (Kelly *et al*., 2018). Single number scores only provide information on whether overall QoL is compromised, but will not yield information regarding which dimension(s) of QoL are impacted and where an individual may need extra support.

Traditional statistical models, such as linear regression, using global or utility scores as the outcome, often fail to reveal underlying heterogeneity in the population. Latent class analysis (LCA), on the other hand, is an unsupervised machine learning analytical technique that uses a top-down approach to capture inter-individual variability to identify latent groups based on observed data (Berlin *et al*., 2014). In psychiatric research, LCA has frequently been applied to determine clinical (Ulbricht *et al*., 2018) and behavioural (Klonsky and Olino, 2008; Foerster and Röösli, 2017) groups, and more recently to understand heterogeneity in QoL among cohorts of people with substance use disorders (De Maeyer *et al*., 2013; Kelly *et al*., 2018). Using these models, it is possible to capture heterogeneity not only in QoL, but to also identify new treatment targets for specific clinical subgroups (e.g., those with low QoL); this moves beyond the notion that the ‘one size fits all’ approach to treatment (Kelly *et al*., 2018).

Although LCA and other clustering analyses have been widely used to understand the heterogeneity of specific disorders (Liao *et al*., 2022), they have rarely been used in general help-seeking clinical populations. Exploring the heterogeneity using QoL in a general help-seeking population, can offer substantial insight into the overall complexity of their social, mental and well-being profiles. This additional information can assist with better funding and resourcing allocation that extend beyond the severity of clinical symptoms. To our knowledge, LCA models have never been applied to understand heterogeneity and complexity in young people seeking help for more common mental health disorders such as depression and anxiety. In general, research is scant on QoL in this population, and hence, we will extend the research in this area.

The overall purpose of this study is to better understand heterogeneity in the QoL of young people at their first presentation to a primary mental health service. Specifically, we aimed to determine: (i) whether distinct and clinically meaningful subgroups of help-seeking young people can be identified based on responses to individual items on the adolescent version of the AQoL-6D using LCA; and (ii) whether and how these groups could be meaningfully discriminated based on demographic and clinical characteristics.

## Method

### Study design

This study was part of a larger study aiming to develop better patient-reported outcome measurement for young people seeking early intervention and treatments for mental ill-health (Filia *et al*., 2021). Institutional ethics approval was obtained from the University of Melbourne Human Research Ethics Committee (1645367.1). Written informed consent was obtained from each young person, and if they were under 18 years, parental/guardian consent was obtained.

### Sample and setting

Young people (aged 12–25 years) were recruited at their first appointment for mental health or substance use related issues in five *headspace* centres across Australia (three metropolitan and two regional). *headspace* is a non-profit organisation established by the Australian Government in 2006, providing accessible, youth-friendly and client-centred primary mental health care to young people aged 12–25 years (McGorry *et al*., 2019; Rickwood *et al*., 2019). Recruitment occurred from September 2016 to April 2018. Data were collected at service entry as well as three-month follow-up.

### Measures

The larger study comprised a total of 18 measures (Filia *et al*., 2021); here we highlight the measures that were pertinent to the current study.

### Self-report measures

#### Quality of life

QoL was assessed using the adolescent version of Assessment of Quality of Life – 6 dimensions, AQoL-6D (Richardson *et al*., 2012). This measure contains 20 items (measured on variable scales with different anchors) in six dimensions, including independent living (4-items; household tasks, mobility outside the home, walking, self-care), social and family relationships (3-items; friendships, family and community role), mental health (4-items; feelings of despair, worry, sadness, tranquillity/agitation), coping (3-items; covering enough energy, being in control, coping with problems), pain (3-items; frequency of pain, severity of pain, degree pain interferes with normal activities) and senses (3-items; seeing, hearing and communication). An example item is ‘*How happy are you with your close and intimate relationships?*’. This item has five possible response choices ranging from 1 ‘*very happy*’ to 5 ‘very unhappy’ (**unweighted rating**). For each dimension, an **unweighted total score** can be derived by summing individual items, with higher scores depicting poorer QoL. The **standardised dimension score** for each dimension was calculated as the reverse min and max scaled unweighted total score with 0 being the ‘worst health state’ and 100 the ‘best health state’ on each dimension. **Total utility scores** were estimated using the published algorithm which comprised individual item weightings for adolescents and ranged from 0 ‘poor QoL’ to 1 ‘good QoL’(Centre for Health Economics, 2014). In Australian adults, the AQoL-6D has been found to have appropriate levels of construct, concurrent and convergent validity (Allen *et al*., 2013). Internal consistency as measured by Cronbach's *α* for most dimensions ranges from 0.73 (coping) to 0.86 (independent living), with lower values for relationships (*α* = 0.63) and senses (*α* = 0.50) dimensions (Allen *et al*., 2013). In our study, the Cronbach's *α* values were comparable with the range of 0.73 (independent living) to 0.93 (pain); relationships and sense dimensions had Cronbach's *α* of 0.64 and 0.54, respectively.

#### Demographic variables

Demographic factors captured included age, sex (at birth), gender, sexual orientation, education and employment status. Lesbian, gay, bisexual, transgender, intersex and queer/questioning (LGBTIQ) status was derived from sex, gender identity and sexual orientation variables. Not in Education, Employment or Training (NEET) status was derived from items pertaining to current education and employment.

#### Clinical symptoms

Clinical symptomatology measurements included the: Patient Health Questionnaire for depression (PHQ-9, scores range from 0 to 27, with higher scores depicting greater symptoms severity) (Kroenke *et al*., 2001; Kroenke and Spitzer, 2002); Generalised Anxiety Disorder-7 item (GAD-7, scores range from 0 to 21, with higher scores indicating greater symptom severity) (Spitzer *et al*., 2006). Although the PHQ-9 and GAD-7 were developed for adults, they have been found to be psychometrically valid in young people (Richardson *et al*., 2010; Mossman *et al*., 2017). Other measures used in this study included the Suicidal Ideation Questionnaire – Junior (SIQ-JR, scores range from 0 to 90 with higher scores indicating worse ideation) for suicidal ideation (Reynolds, 1987) and the Pittsburgh Sleep Quality Index (only self-rated questions scored with scores ranging from 0 to 21, higher scores indicate more problematic sleep) (Buysse *et al*., 1989).

### Clinician and interviewer ratings

Diagnoses were formulated by clinicians based on the Diagnostic and Statistical Manual of Mental Disorders – Fifth Edition (DSM5) (American Psychiatric Association, 2013). Research assistants extracted these diagnoses from medical files. The clinical staging model developed by McGorry *et al*. (2006), was used to assess the severity and progression of participants' psychiatric symptoms. Research assistants rated the clinical stage on a scale from 0 to 4: increased risk without symptoms (stage 0); mild or non-specific symptoms (stage 1a); ultra-high risk (stage 1b); and full threshold or above (stages 2–4).

### Statistical analyses

All statistical analyses were conducted in **R version 4.0.3** (R Core Team, 2020). Detailed statistical methods and justifications are provided in the Supplementary Material and a summary is included below.

#### Distribution and structure of AQoL-6D items

Bar plots and histogram plots were used to visualise the distributions of **unweighted individual items** as well as **standardised dimension scores**. A multidimensional scaling (MDS) network plot (Jones *et al*., 2018) was used to understand whether the individual items within a dimension were measuring the same latent construct depicted by that dimension. Based on polychoric correlation coefficients (*r_pc_*), this plot indicates that the closer the nodes (items), then the greater the degree of association between items.

#### Latent class analysis

LCA based on **unweighted individual items** (modelled with multinomial distribution) was used to empirically evaluate the possible heterogeneous groups. To improve model stability, 10-fold cross-validation (CV), leave-one-site-out (LOSO) CV and split-half CV were used to identify the best number of classes instead of the traditional log-likelihood ratio test (Payne *et al*., 2011; Grimm *et al*., 2017), see Supplementary Material. Bayesian information criterion (BIC) was used as the main fitting index for choosing class numbers (Nylund *et al*., 2007) with Akaike information criteria (AIC) and log-likelihood guiding interpretation. Sensitivity analyses were conducted using k-means clustering based on principal components.

### Profiles of latent classes

To validate class differences, we first compared the distribution of AQoL-6D **standardised dimension** and **total utility scores**. Descriptive statistics were used to compare differences in risk factors between classes. Risk factors that could explain observed heterogeneity were identified from the study's wider assessment battery and included demographic variables, diagnosis, clinical staging, PHQ-9, GAD-7; SIQ-Jnr and PSQI. Multivariate multinomial logistic regression models were subsequently carried out to further validate LCA results (also known as the 3-step LCA method) and identify risk factors that could best predict class membership. Two separate models were used due to possible overlap between clinical diagnosis/severity and self-reported clinical symptoms (demographic variables, diagnosis and clinical staging included in the first model and the second model additionally included clinical symptoms). Relative risk ratios (RRR) are reported, which represent the risk of the outcome falling in one outcome group relative to the reference outcome group with an increase or presence of a given risk factor while controlling for the effect of other risk factors.

## Results

### Cohort characteristics

The cohort has previously been described elsewhere (Filia *et al*., 2021). The overall cohort comprised 1107 individuals; however, only 1067 had complete information from the AQoL-6D and were included in the analyses. The demographic and clinical characteristics of the 1067 can be found in Table 1. Briefly, the median age was 18 (IQR [16–20]), 65% of the cohort were female, and 63% were attending a *headspace* service in a metropolitan region. The most common diagnosis was depression and anxiety (33%) followed by only anxiety (26%).

**Table 1.** Demographic and clinical characteristics of participants by the four identified latent classes

Characteristic^a^	Overall, *N* = 1067^b^	No/Mild, *n* = 280^a^	Moderate-Phy, *n* = 296^a^	Moderate-Psy, *n* = 304^a^	Severe, *n* = 187^a^
Age in years	18 (16, 20)	17 (14, 20)	18 (15, 20)	19 (17, 21)	19 (16, 21)
Age group					
Age 12–17	476 (45%)	155 (55%)	148 (50%)	106 (35%)	67 (36%)
Age 18–25	591 (55%)	125 (45%)	148 (50%)	198 (65%)	120 (64%)
Sex at birth					
Male	375 (35%)	148 (53%)	107 (36%)	83 (27%)	37 (20%)
Female	692 (65%)	132 (47%)	189 (64%)	221 (73%)	150 (80%)
LGBTIQ	297 (29%)	46 (17%)	93 (33%)	83 (28%)	75 (41%)
Region					
Metro	671 (63%)	182 (65%)	168 (57%)	201 (66%)	120 (64%)
Regional	396 (37%)	98 (35%)	128 (43%)	103 (34%)	67 (36%)
NEET	158 (15%)	36 (13%)	38 (13%)	40 (14%)	44 (24%)
Primary diagnosis					
Anxiety	264 (26%)	81 (30%)	70 (25%)	79 (28%)	34 (20%)
Depression	182 (18%)	38 (14%)	62 (22%)	50 (18%)	32 (18%)
Depression and anxiety	331 (33%)	51 (19%)	88 (31%)	118 (41%)	74 (43%)
Other^c^	237 (23%)	101 (37%)	64 (23%)	38 (13%)	34 (20%)
Clinical staging					
0–1a	625 (60%)	216 (78%)	184 (63%)	170 (58%)	55 (31%)
1b	326 (31%)	53 (19%)	87 (30%)	102 (35%)	84 (47%)
2–4	85 (8.2%)	7 (2.5%)	19 (6.6%)	21 (7.2%)	38 (21%)
PHQ-9	13 (8, 18)	6 (3, 9)	13 (9, 16)	14 (11, 17)	21 (18, 24)
GAD-7	10 (6, 14)	5 (2, 7)	10 (7, 13)	12 (8, 15)	17 (14, 19)
SIQ-Jr	12 (4, 28)	4 (0, 10)	14 (8, 29)	14 (7, 28)	40 (16, 59)
PSQI	8 (5, 11)	5 (4, 8)	8 (6, 11)	8 (6, 11)	12 (9, 14)

a Missing data include 42 for LGBTIQ, 32 for NEET, 53 for primary diagnosis, 31 for clinical staging, 4 for PHQ-9, 6 for GAD-7, 6 for SIQ-Jr and 48 for PSQI.

b Statistics presented: Median (IQR); *n* (%).

c Other diagnoses.

### Profile of quality of life in the cohort

Distributions of individual AQoL-6D items are displayed in online Supplementary Fig. A1 (Supplementary Materials). Poorer QoL (higher scores) can be observed for many of the items, in particular for individual items pertaining to mental health and coping. For coping and mental health dimensions (based on standardised scores of 0 ‘worst QoL’ to 100 ‘best QoL’) distributions were relatively normal and average scores in these dimensions indicated greater levels of impairment compared to the other four dimensions (see online Supplementary Fig. A2).

**Fig. 1.**
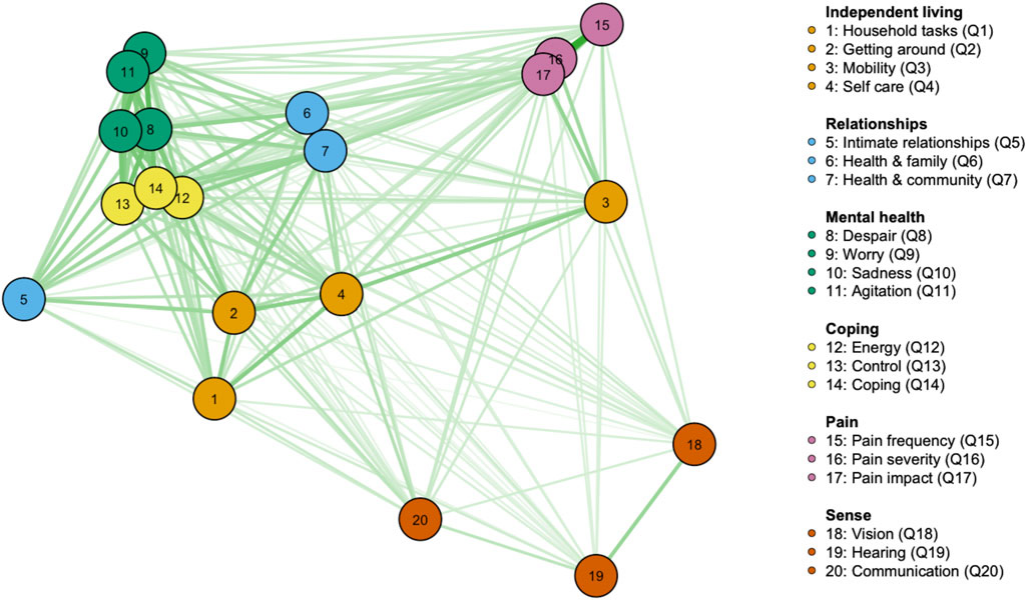
Polychoric Correlation (Rpc) Network of AQoL-6D Items.

The inter-item *r_pc_* correlation matrix is shown in online Supplementary Fig. A3. Correlations ranged from 0.06 (between Q5 ‘How happy do your close relationships make you?’ and Q18 ‘How good is your vision?’) to 0.83 (between Q16 ‘How much physical pain or discomfort do you experience?’ and Q17 ‘How does pain interfere with your usual activities?’). Figure 1 is the network plot of pairwise *r_pc_* inter-item correlations; all items were positively associated with each other. Clusters of items are apparent for most dimensions (higher internal correlations).

### Latent class analysis

A range of models was run from the training data (10-sets for 10-fold CV and 5-sets for LOSO CV), see fitting indices in online Supplementary Figs A4 and A5. CV results indicate that the model with four latent classes was the best fitting model. BIC was lowest at 4-class model and AIC and log-likelihood also show an elbow point at 4-class model, suggesting little improvement in model fitting with increasing class numbers. Therefore, a 4-class model was chosen to be the best model and was then fitted in the total cohort. Both split-half CV and k-means clustering further validated the identified classes (results not shown).

### Profile of latent class memberships

**Fig. 2.**
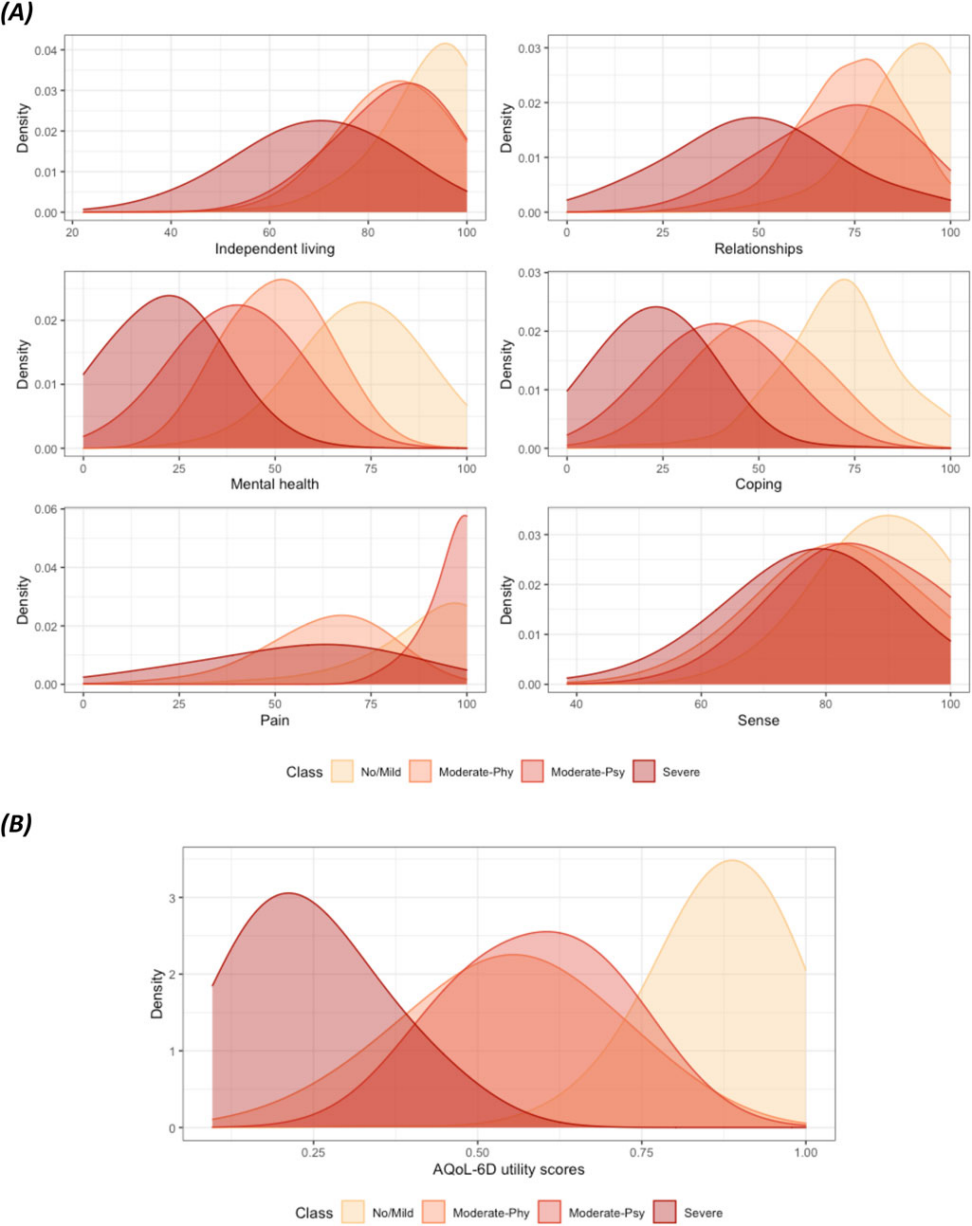
Distributions of the (A) Standardised AQoL-6D Dimension Scores (B) Total Utility Scores by Identified Latent Class Membership.

Class differences on standardised dimension scores and total utility scores can be found in Figs 2A and 2B and in online Supplementary Table A1. The first latent class, ‘No/Mild’, had no to very mild impairments in QoL, and higher ratings across QoL dimensions. The second and third classes were similar in independent living, relationships and sense, but one group showed higher impairment in pain and the other in mental health and coping dimensions. Hence, we named these groups ‘Moderate-Phy’ and ‘Moderate-Psy’. The last identified latent class, labelled as ‘Severe’, showed greater levels of impairment in QoL across all dimensions compared with other groups. The mean utility score of the ‘Severe’ group was only 0.25 (s.d. = 0.10), compared with a mean of 0.87 (s.d. = 0.10) in the ‘No/Mild’ group, which had values commensurate to the population norms (Maxwell *et al*., 2016). The mean utility score of the ‘Moderate-Phy’ group was slightly lower than the ‘Moderate-Psy’ group, 0.54 (s.d. = 0.14) vs. 0.59 (s.d. = 0.12).

Table 1 depicts the demographic, social and clinical profiles of four classes. Compared with other groups, the ‘Severe’ group represented the most severe or complex subgroup, and included more participants who were older, female, LGBTIQ, with NEET status, with a diagnosis of, and more severe, anxiety and depression, as well as a clinical staging of ultra-high risk (stage 1b) or a full-threshold diagnosed psychiatric disorder (stages 2–4). This group also had poorer sleep and more suicidal thoughts (SIQ-JR *Mdn* *=* 40 compared with *Mdn* *=* 4 in the ‘No/Mild’ group).

The ‘Moderate-Phy’ and ‘Moderate-Psy’ groups were largely comparable; however, the ‘Moderate-Phy’ group had a higher proportion of young people aged between 12 and 17 years, belonging to the LGBTIQ community, and with a primary diagnosis other than anxiety and/or depression. The Moderate-Phy group comprised individuals with provisional diagnoses (*n* = 30) and disorders including personality (*n* = 26), adjustment (*n* = 24), substance use (*n* = 17), behavioural (*n* = 17), psychosis (*n* = 4) and other (i.e., developmental disorders, *n* = 119). Those in the ‘Moderate-Psy’ group were more likely to be female, and had more severe depression and anxiety.

Results from the first multinomial multivariate logistic regression model (without self-reported symptoms) are displayed in Table 2. The vast majority of the univariate associations were also retained in the multivariate model. A trend of increasing severity and complexity was observed from the ‘No/Mild’ to the ‘Severe’ group. The relative risk for being in the full-threshold staging (2–4) relative to stages of increased risk and without specific symptoms (0–1a) was estimated over 14 (RRR: 14.56; 95% CI: 5.80–36.53) times higher in the ‘Severe’ group versus the ‘No/Mild’ group. When controlling for self-reported symptoms (shown in Table 3), the RR was still over 7 times higher compared with the ‘No/Mild’ group (RRR 7.20; 95% CI 1.93–26.90) and over 2 times higher compared with the ‘Moderate-Psy’ (RRR: 2.75; 95%CI: 1.16–6.49) and ‘Moderate-Phy’ group (RRR: 3.12, 95% CI: 1.25–7.78). The ‘Severe’ group had higher levels of clinical symptoms independently across multiple domains including depression, anxiety, suicidal thoughts and sleep quality. More severe self-reported clinical symptoms, particularly depressive symptoms, were found in both the ‘Moderate’ and ‘Severe’ groups relative to the ‘No/Mild’ group. Compared with the ‘Moderate-Phy’ group, the ‘Moderate-Psy’ group was older (RRR: 1.11 95%CI: 1.05–1.18), female (RRR: 1.53; 95% CI: 1.05–2.21), and have a higher level of depression (RRR: 1.33; 95%CI: 0.99–1.80) and anxiety (RRR:1.38; 95%CI: 1.08–1.75); whereas, the ‘Moderate-Phy’ group were more likely to be from regional areas (RRR: 1.73, 95%CI: 1.21–2.46).

**Table 2.** Multinomial logistic regression results (imputed)

	Moderate-Phy vs No/Mild		Moderate-Psy vs No/Mild		Severe vs No/Mild	
	RRR (95% CI)	*p*-value	RRR (95% CI)	*p*-value	RRR (95% CI)	*p*-value
Age in years	1.01 (0.95–1.06)	0.836	1.11 (1.05–1.18)	<0.001	1.06 (0.99–1.13)	0.096
Sex at birth						
Male	Ref		Ref		Ref	
Female	1.82 (1.29–2.58)	<0.001	3.00 (2.07–4.35)	<0.001	4.69 (2.92–7.53)	<0.001
LGBTIQ						
No	Ref		Ref		Ref	
Yes	1.76 (1.16–2.66)	0.007	1.29 (0.84–1.99)	0.245	1.86 (1.15–2.99)	0.011
Region						
Metro	Ref		Ref		Ref	
Regional	1.29 (0.90–1.85)	0.162	0.74 (0.51–1.08)	0.115	0.74 (0.47–1.15)	0.181
NEET						
No	Ref		Ref		Ref	
Yes	0.91 (0.54–1.55)	0.733	0.92 (0.54–1.56)	0.748	1.63 (0.92–2.89)	0.095
Primary diagnosis						
Anxiety	Ref		Ref		Ref	
Depression	1.68 (0.99–2.86)	0.054	1.31 (0.75–2.30)	0.348	1.54 (0.79–3.00)	0.206
Depression and Anxiety	1.84 (1.14–2.98)	0.012	2.08 (1.28–3.36)	0.003	2.37 (1.32–4.24)	0.004
Other	0.74 (0.47–1.18)	0.211	0.43 (0.26–0.71)	<0.001	0.61 (0.33–1.12)	0.110
Clinical staging						
0–1a	Ref		Ref		Ref	
1b	1.56 (1.03–2.38)	0.038	2.11 (1.39–3.20)	<0.001	4.75 (2.94–7.68)	<0.001
2–4	3.09 (1.22–7.82)	0.017	3.44 (1.36–8.69)	0.009	14.56 (5.80–36.53)	<0.001

*RRR represents relative risk ratio associated with one standard deviation change in the risk factor. All clinical variables were standardised. Age was not standardised. Standardisation was not needed for dummy variables. Cohort s.d.s are: 6.6 for PHQ-9; 5.7 for GAD-7; 20.3 for SIQ-JR; 3.8 for PSQI. The average prediction accuracy from imputed models is 0.413.

**Table 3.** Multinomial logistic regression results including clinical outcomes as risk factors (imputed)

	Moderate-Phy vs No/Mild		Moderate-Psy vs No/Mild		Severe vs No/Mild	
	RRR (95% CI)	*p*-value	RRR (95% CI)	*p*-value	RRR (95% CI)	*p*-value
Age in years	1.00 (0.93–1.07)	0.976	1.11 (1.04–1.19)	0.003	1.07 (0.97–1.18)	0.206
Sex at birth						
Male	Ref		Ref		Ref	
Female	1.49 (0.97–2.30)	0.072	2.28 (1.43–3.63)	<0.001	3.53 (1.78–6.99)	<0.001
LGBTIQ						
No	Ref		Ref		Ref	
Yes	1.37 (0.82–2.28)	0.230	1.01 (0.59–1.74)	0.960	0.89 (0.45–1.78)	0.751
Region						
Metro	Ref		Ref		Ref	
Regional	1.41 (0.91–2.19)	0.121	0.82 (0.51–1.31)	0.406	0.80 (0.43–1.52)	0.504
NEET						
No	Ref		Ref		Ref	
Yes	1.12 (0.59–2.11)	0.730	1.09 (0.56–2.15)	0.794	2.07 (0.88–4.85)	0.094
Primary diagnosis						
Anxiety	Ref		Ref		Ref	
Depression	1.42 (0.73–2.76)	0.298	1.23 (0.61–2.50)	0.559	1.61 (0.63–4.12)	0.320
Depression and Anxiety	1.46 (0.82–2.60)	0.199	1.79 (1.00–3.20)	0.051	1.66 (0.75–3.68)	0.215
Other	1.14 (0.64–2.05)	0.655	0.72 (0.37–1.38)	0.323	0.96 (0.39–2.38)	0.932
Clinical staging						
0–1a	Ref		Ref		Ref	
1b	0.66 (0.39–1.12)	0.127	0.83 (0.48–1.44)	0.511	1.00 (0.49–2.04)	0.998
2–4	2.31 (0.76–7.05)	0.142	2.62 (0.84–8.18)	0.098	7.20 (1.93–26.90)	0.003
PHQ-9^a^	2.76 (1.87–4.06)	<0.001	3.67 (2.44–5.52)	<0.001	16.64 (9.28–29.84)	<0.001
GAD-7^a^	2.17 (1.58–2.97)	<0.001	2.98 (2.14–4.15)	<0.001	6.34 (4.05–9.92)	<0.001
SIQ-Jr^a^	2.24 (1.52–3.30)	<0.001	1.99 (1.33–2.97)	<0.001	2.50 (1.60–3.93)	<0.001
PSQI^a^	1.59 (1.18–2.14)	0.002	1.37 (1.00–1.89)	0.053	2.25 (1.51–3.35)	<0.001

a RRR represents relative risk ratio associated with one standard deviation change in the risk factor. All clinical variables were standardised. Age was not standardised, and standardisation was not needed for dummy variables Cohort s.d.s are: 6.6 for PHQ-9; 5.7 for GAD-7; 20.3 for SIQ-JR; 3.8 for PSQI. The average prediction accuracy from imputed models is 0.581.

## Discussion

QoL is an important outcome from the perspective of young people and their families (Ravens-Sieberer *et al*., 2014). Not much is known about QoL in young people presenting to mental health services and QoL instruments are rarely routinely collected by these services. In this novel study, we investigated the heterogeneity in young people presenting to primary mental health care. Importantly, we identified four distinct groups of young people based on their responses to the AQoL-6D and these groups were externally validated based on demographic and clinical characteristics. The latent subgroups reflect varying levels of complexity (e.g., functioning, physical health) which would not necessarily be identified using clinical symptom measures such as psychological distress. Young people presented with heterogeneities in both the severity and types of impairments in QoL, which reflects their diverse needs in care type and intensity. This approach offers valuable holistic insights that have the potential to improve the targeting of primary mental health care services for young people with specific care needs.

The group with ‘No/Mild’ QoL impairments is likely to have distinct care and resource needs compared to the three other QoL groups. The ‘No/Mild’ group had health utility scores comparable with age-matched peers in the general population (Maxwell *et al*., 2016). This group were younger (especially compared to those in the ‘Moderate-Psy’ group), more likely to be male, less likely to have a diagnosis of a mood and/or anxiety disorder, have less severe symptomatology and suicidal ideation, and to be in the early stages of illness course. Preventing worsening of symptoms and chronicity, managing comorbidities and minimising functional decline, would all be useful targets in preventing the decline of QoL in this group.

The ‘Severe’ group showed impaired QoL across all AQoL-6D dimensions with a mean utility score less than 30% of those reported by the ‘No/Mild’ group. This ‘Severe’ group seemed to have a complex presentation including both anxiety and depressive disorder diagnoses, more severe symptomatology, suicidal ideation and poorer sleep. Female sex and those with LGBTIQ status were over-represented in this group. Some of these factors could be considered risk factors for both poor QoL and mental health issues. For example, young people who identify as LGBTIQ often encounter distinct challenges in identity development and social acceptance (Brown *et al*., 2016). Because of these issues, they are more likely to report poorer QoL (Charlton *et al*., 2018) and mental health (Higgins *et al*., 2021). Their mental health problems are also likely to impact their QoL (Bosse, 2019). Mental health services need to understand such challenges in order to improve help-seeking and treatment engagement (Brown *et al*., 2016).

We also defined two groups with moderate impairments in QoL. While the groups did not differ with respect to suicidality or sleep quality, the ‘Moderate-Psy’ group had higher levels of affective symptoms, and were more likely to be female than the ‘Moderate-Phy’ group. This might be due to affective and emotional problems being more commonly reported by females (Zahn-Waxler *et al*., 2008).

The ‘Moderate-Phy’ group are of particular interest. They were more likely to have received services from regional clinics, have other diagnoses and were more likely to report pain. There are a number of reasons as to why this may be the case. First, adolescence is a developmental period where somatic pain can manifest and predominate. In a large WHO-based study of pain in adolescents (up to 18 years of age), around 50% reported headaches and/or stomach pain and nearly 40% reported backache; 35.7% of young people experienced all three types of pain (Swain *et al*., 2014). Pain onset often precedes mental disorders, but it is expected the relationship is bidirectional (Slater *et al*., 2016). Pain, depression and anxiety can also have shared biological pathways (Simons *et al*., 2014). Second, young people in regional areas, might have concerns about confidentiality and stigma when accessing mental health services, and might present with physical rather than mental ill-health. Finally, finding that the two moderate groups did not differ in suicidality highlights the impact of pain on young people can be just as much as anxiety and depression. Further research, however, is needed to more closely look at the needs of these young people and how to best support them. There is a lack of developmentally-appropriate resources available to support this group and they may be missed in both primary health and mental health settings (Slater *et al*., 2016).

Most of the AQoL-6D dimensions seem to differentiate between the latent subgroups identified except for the sense domain on physical impairment in vision, hearing and communication. This is potentially explained by the lack of participation of physically disabled young people in the study. One may question where these young people receive support for mental ill-health and what barriers they may encounter in accessing services.

These findings have a number of important implications. First, young people with mental health issues have varied perceptions of their QoL. Third, these varied perceptions are associated with different demographic and clinical characteristics, and highlight varying degrees of complexity when young people first present to mental health services. Third, just obtaining information about symptoms or focusing on a total score on a QoL scale may mean that important aspects of a young person's life are missed, and such factors can be integral to treatment recovery. Finally, self-reported QoL measures can offer a cost-effective and subjective overview of an individual's life satisfaction across multiple domains and degrees of impairment. Having a young person complete such a measure, may be helpful for guiding focus in clinical assessments and alerting clinicians to potential risk factors that can be associated with poorer outcomes.

### Broader implications

These findings have implications for service planners, clinical researchers and economic evaluators. Integrated youth health services, such as *headspace,* are a focus of mental health reforms in a number of countries and are typically designed to provide youth-friendly services for young people with mild-to-moderate or subthreshold symptoms; however, it has been noted that there is a growing frequency of young people with full-threshold diagnoses and complex presentations (Rickwood *et al*., 2014). But symptoms and diagnoses are not the only features of complex presentations and QoL may help better tailor service responses within primary mental health care. Measures such as the AQoL-6D, may provide useful information about a young person's standing, over and above psychological indices such as the PHQ-9. For example, in mental health services, information about physical health and/or experiences of pain are not routinely collected, and poorer physical QoL might be an important contributor to the severity of the young person's depressive symptoms. Internalising disorders are often worse in adolescents experiencing pain than those not experiencing pain (Noel *et al*., 2016). Pain, however, can often go under recognised or misdiagnosed in young people (Friedrichsdorf *et al*., 2016). Resourcing clinicians in how to assess for, and manage pain in young people presenting to mental health services should be considered.

Our findings also have a number of implications for those undertaking or interpreting economic studies in primary youth mental health care. Notably, we have shown it is possible to identify groups who are likely to have very different patterns of costs and benefits. As the ‘No/Mild’ group has equivalent health utility scores to population norms, the scope for health utility gain is small, and the cost-effectiveness of many interventions for this group (who are younger and at earlier clinical stages) may depend on averting future ill-health and arresting illness progression. By contrast, the utility loss in the ‘Severe’ group is substantial and the older age, increased likelihood of being disengaged from work and study, and heightened suicide risk suggest that productivity loss in this group may also be high. Interventions that are effective at addressing these issues may prove cost-effective even if relatively resource intensive. Finally, for some in the ‘Moderate-Phy’ group, a question worth exploring is whether mental and/or physical health interventions may be the most feasible and cost-effective options for achieving utility gain. In this respect, economic evaluations of the design of incentives to ensure adequate participation of general practitioners and potential for inclusion of physiotherapists in youth mental health clinics may be warranted. This suggests that economic researchers should evaluate primary youth mental health services as complex systems in which the cost-effectiveness of service delivery may vary across different client groups.

### Limitations

A range of factors (e.g., sample size for the total cohort and for some of the classes such as ‘Severe’ group, local dependence, high dimensionality and rare outcome groups) may impact the integrity of the LCA models (Swanson *et al*., 2012). CV methods applied in the study only assert the validity within the study sample, and future external validation is needed to identify whether findings can be replicated in other independent samples.

### Future research

There are a lot of scopes to extend this novel work. Given QoL measures differ in dimensionality, it would be interesting to see whether the latent classes using the AQoL-6D can be validated using other QoL scales. There have been other studies that have used measures such as the Eurohis-QoL (EQoL) (Kelly *et al*., 2018) and the WHOQoL (Liao *et al*., 2022) in other populations (substance use and first-episode psychosis, respectively) who have found only three clusters. Do the latent classes differ according to the items covered by the QoL scale and the population under examination?

The current study was an important first step in understanding heterogeneity in QoL in young people with mental health issues; however, the focus was on cross-sectional data. To extend this work it would be of interest to determine the temporal stability of these groups over time. To add weight to the clinical meaningfulness of these groups, it would be also worthwhile to determine whether these groups differ in the trajectory of symptoms and functioning over the course of treatment, and the level of young people's treatment engagement using contemporary modelling techniques. For example, as an extension to latent class models, the Hidden Markov modelling technique (Rabiner and Juang, 1986), can be used to evaluate whether and how individuals transit between these severity groups in longitudinal settings and evaluate risk factors associated with recovery in QoL.

## Conclusions

We have shown that it is possible to identify meaningful groups of young people seeking help for mental ill-health based on their QoL. As a standardised measure to add value to direct a holistic biopsychosocial assessment, adding multi-attribute utility instruments such as the AQoL-6D to routine data collection in mental health services has the potential to generate insights that may improve the provision and targeting of care for young people. In young people with impairments across all QoL dimensions, the need for a holistic and personalised approach to treatment and recovery is particularly important.

## Supplementary material

For supplementary material accompanying this paper visit https://doi.org/10.1017/S2045796022000427. click here to view supplementary material

## Availability of data and materials

The datasets used in the current study are available from the corresponding author on reasonable request. A synthetically generated dataset and a R package developed for future use of our analytical method are available on the GitHub repository: https://ready4-dev.github.io/heterodox/

## References

[ref1] Aaronson NK (1988) Quantitative issues in health-related quality of life assessment. Health Policy 10, 217–230. 10291115 10.1016/0168-8510(88)90058-9

[ref2] Allen J, Inder KJ, Lewin TJ, Attia JR and Kelly BJ (2013) Construct validity of the Assessment of Quality of Life – 6D (AQoL-6D) in community samples. Health and Quality of Life Outcomes 11, 61. 23590808 10.1186/1477-7525-11-61 PMC3639231

[ref3] American Psychiatric Association (2013) Diagnostic and Statistical Manual of Mental Disorders. Fifth Edition (DSM-5). Washington, DC: American Psychiatric Association.

[ref4] Berlin KS, Williams NA and Parra GR (2014) An introduction to latent variable mixture modeling (part 1): overview and cross-sectional latent class and latent profile analyses. Journal of Pediatric Psychology 39, 174–187. 24277769 10.1093/jpepsy/jst084

[ref5] Bosse JD (2019) Sexual and gender identity development in young adults and implications for healthcare. Current Sexual Health Reports 11, 274–286.

[ref6] Brown A, Rice SM, Rickwood DJ and Parker AG (2016) Systematic review of barriers and facilitators to accessing and engaging with mental health care among at-risk young people. Asia-Pacific Psychiatry 8, 3–22. 26238088 10.1111/appy.12199

[ref7] Bullinger M and Quitmann J (2014) Quality of life as patient-reported outcomes: principles of assessment. Dialogues in Clinical Neuroscience 16, 137–145. 25152653 10.31887/DCNS.2014.16.2/mbullinger PMC4140508

[ref8] Buysse DJ, Reynold CF, Monk TH, Berman SR and Kupfer DJ (1989) The Pittsburgh Sleep Quality Index: a new instrument for psychiatric practice and research. Psychiatry Research 28, 193–213. 2748771 10.1016/0165-1781(89)90047-4

[ref9] Centre for Health Economics (2014) AQoL-6D Adolescent algorithm SPSS. Available at: http://www.aqol.com.au/index.php/scoring-algorithms?id=201 (Accessed 1 February 2021).

[ref10] Charlton BM, Gordon AR, Reisner SL, Sarda V, Samnaliev M and Austin SB (2018) Sexual orientation-related disparities in employment, health insurance, healthcare access and health-related quality of life: a cohort study of US male and female adolescents and young adults. BMJ Open 8, e020418. 10.1136/bmjopen-2017-020418 PMC6067349 30049672

[ref11] De Maeyer J, van Nieuwenhuizen C, Bongers IL, Broekaert E and Vanderplasschen W (2013) Profiles of quality of life in opiate-dependent individuals after starting methadone treatment: a latent class analysis. The International Journal on Drug Policy 24, 342–350. 23127664 10.1016/j.drugpo.2012.09.005

[ref12] EuroQol group (1990) EuroQol--a new facility for the measurement of health-related quality of life. Health Policy 16, 199–208. 10109801 10.1016/0168-8510(90)90421-9

[ref13] Filia K, Rickwood D, Menssink J, Gao CX, Hetrick S, Parker A, Hamilton M, Hickie I, Herrman H, Telford N, Sharmin S, McGorry P and Cotton S (2021) Clinical and functional characteristics of a subsample of young people presenting for primary mental healthcare at headspace services across Australia. Social Psychiatry and Psychiatric Epidemiology 56, 1311–1323. 33452888 10.1007/s00127-020-02020-6

[ref14] Foerster M and Röösli M (2017) A latent class analysis on adolescents media use and associations with health related quality of life. Computers in Human Behavior 71, 266–274.

[ref15] Friedrichsdorf SJ, Giordano J, Desai Dakoji K, Warmuth A, Daughtry C and Schulz CA (2016) Chronic pain in children and adolescents: diagnosis and treatment of primary pain disorders in head, abdomen, muscles and joints. Children 3, 42. 10.3390/children3040042 PMC5184817 27973405

[ref16] Grimm KJ, Mazza GL and Davoudzadeh P (2017) Model selection in finite mixture models: a k-fold cross-validation approach. Structural Equation Modeling 24, 246–256.

[ref17] Hansell NK, Wright MJ, Medland SE, Davenport TA, Wray NR, Martin NG and Hickie IB (2012) Genetic co-morbidity between neuroticism, anxiety/depression and somatic distress in a population sample of adolescent and young adult twins. Psychological Medicine 42, 1249–1260. 22051348 10.1017/S0033291711002431

[ref18] Hawthorne G, Richardson J and Osborne R (1999) The Assessment of Quality of Life (AQoL) instrument: a psychometric measure of Health-Related Quality of Life. Quality of Life Research 8, 209–224. 10472152 10.1023/a:1008815005736

[ref19] Hickie IB, Scott EM, Cross SP, Iorfino F, Davenport TA, Guastella AJ, Naismith SL, Carpenter JS, Rohleder C, Crouse JJ, Hermens DF, Koethe D, Markus Leweke F, Tickell AM, Sawrikar V and Scott J (2019) Right care, first time: a highly personalised and measurement-based care model to manage youth mental health. The Medical Journal of Australia 211(suppl. 9), S3–S46. 10.5694/mja2.50383 31679171

[ref20] Higgins A, Downes C, Murphy R, Sharek D, Begley T, McCann E, Sheerin F, Smyth S, De Vries J and Doyle L (2021) LGBT + young people's perceptions of barriers to accessing mental health services in Ireland. Journal of Nursing Management 29, 58–67. 33068465 10.1111/jonm.13186

[ref21] Higginson IJ and Carr AJ (2001) Measuring quality of life: using quality of life measures in the clinical setting. BMJ 322, 1297–1300. 11375237 10.1136/bmj.322.7297.1297 PMC1120388

[ref22] Jones PJ, Mair P and McNally RJ (2018) Visualizing psychological networks: a tutorial in R. Frontiers in Psychology 9, 1742. 30283387 10.3389/fpsyg.2018.01742 PMC6156459

[ref23] Kelly PJ, Robinson LD, Baker AL, Deane FP, Osborne B, Hudson S and Hides L (2018) Quality of life of individuals seeking treatment at specialist non-government alcohol and other drug treatment services: a latent class analysis. Journal of Substance Abuse Treatment 94, 47–54. 30243417 10.1016/j.jsat.2018.08.007

[ref24] Klonsky ED and Olino TM (2008) Identifying clinically distinct subgroups of self-injurers among young adults: a latent class analysis. Journal of Consulting and Clinical Psychology 76, 22–27. 18229979 10.1037/0022-006X.76.1.22

[ref25] Kroenke K and Spitzer RL (2002) The PHQ-9: a new depression diagnostic and severity measure. Psychiatric Annals 32, 1–7.

[ref26] Kroenke K, Spitzer RL and Williams JB (2001) The PHQ-9: validity of a brief depression severity measure. Journal of General Internal Medicine 16, 606–613. 11556941 10.1046/j.1525-1497.2001.016009606.x PMC1495268

[ref27] Liao Z, Allott K, Anderson JFI, Killackey E and Cotton SM (2022) Quality of life in first-episode psychosis: a cluster analytic approach. Quality of Life Research 31, 1807–1817. 10.1007/s11136-021-03014-w. 34661805

[ref28] Maxwell A, Özmen M, Iezzi A and Richardson J (2016) Deriving population norms for the AQoL-6D and AQoL-8D multi-attribute utility instruments from web-based data. Quality of Life Research 25, 3209–3219. 27344318 10.1007/s11136-016-1337-z

[ref29] McGorry PD, Hickie IB, Yung AR, Pantelis C and Jackson HJ (2006) Clinical staging of psychiatric disorders: a heuristic framework for choosing earlier, safer and more effective interventions. Australian and New Zealand Journal of Psychiatry 40, 616–622. 16866756 10.1080/j.1440-1614.2006.01860.x

[ref30] McGorry P, Trethowan J and Rickwood D (2019) Creating headspace for integrated youth mental health care. World Psychiatry 18, 140–141. 31059618 10.1002/wps.20619 PMC6502425

[ref31] Mossman SA, Luft MJ, Schroeder HK, Varney ST, Fleck DE, Barzman DH, Gilman R, DelBello MP and Strawn JR (2017) The Generalized Anxiety Disorder 7-item scale in adolescents with generalized anxiety disorder: signal detection and validation. Annals of Clinical Psychiatry 29, 227–234A. 29069107 PMC5765270

[ref32] Noel M, Groenewald CB, Beals-Erickson SE, Gebert JT and Palermo TM (2016) Chronic pain in adolescence and internalizing mental health disorders: a nationally representative study. Pain 157, 1333–1338. 26901806 10.1097/j.pain.0000000000000522 PMC4939835

[ref33] Nylund KL, Asparouhov T and Muthén BO (2007) Deciding on the number of classes in latent class analysis and growth mixture modeling: a Monte Carlo simulation study. Structural Equation Modeling: A Multidisciplinary Journal 14, 535–569.

[ref34] Payne RJ, Telford RJ, Blackford JJ, Blundell A, Booth RK, Charman DJ, Lamentowicz Ł, Lamentowicz M, Mitchell EAD, Potts G, Swindles GT, Warner BG and Woodland W (2011) Testing peatland testate amoeba transfer functions: appropriate methods for clustered training-sets. The Holocene 22, 819–825.

[ref35] Rabiner L and Juang B (1986) An introduction to hidden Markov models. IEEE ASSP Magazine 3, 4–16.

[ref36] Ravens-Sieberer U, Karow A, Barthel D and Klasen F (2014) How to assess quality of life in child and adolescent psychiatry. Dialogues in Clinical Neuroscience 16, 147–158. 25152654 10.31887/DCNS.2014.16.2/usieberer PMC4140509

[ref37] R Core Team (2020) A Language and Environment for Statistical Computing. R Foundation for Statistical Computing, 4.0.3 Edn. Vienna, Austria: R Foundation for Statistical Computing.

[ref38] Reynolds WM (1987) Suicidal Ideation Questionnaire (SIQ): Professional Manual. Odessa, Florida: Psychological Assessment Resources.

[ref39] Richardson LP, McCauley E, Grossman DC, McCarty CA, Richards J, Russo JE, Rockhill C and Katon W (2010) Evaluation of the Patient Health Questionnaire-9 Item for detecting major depression among adolescents. Pediatrics 126, 1117–1123. 21041282 10.1542/peds.2010-0852 PMC3217785

[ref40] Richardson JR, Peacock SJ, Hawthorne G, Iezzi A, Elsworth G and Day NA (2012) Construction of the descriptive system for the Assessment of Quality of Life AQoL-6D utility instrument. Health and Quality of Life Outcomes 10, 38. 22507254 10.1186/1477-7525-10-38 PMC3349491

[ref41] Rickwood DJ, Telford NR, Parker AG, Tanti CJ and McGorry PD (2014) *headspace* – Australia's innovation in youth mental health: who are the clients and why are they presenting. Medical Journal of Australia 200, 108–111. 24484115 10.5694/mja13.11235

[ref42] Rickwood D, Paraskakis M, Quin D, Hobbs N, Ryall V, Trethowan J and McGorry P (2019) Australia's innovation in youth mental health care: the headspace centre model. Early Intervention in Psychiatry 13, 159–166. 30311423 10.1111/eip.12740 PMC6585724

[ref43] Sawyer MG, Whaites L, Rey JM, Hazell PL, Graetz BW and Baghurst P (2002) Health-related quality of life of children and adolescents with mental disorders. Journal of the American Academy of Child and Adolescent Psychiatry 41, 530–537. 12014785 10.1097/00004583-200205000-00010

[ref44] Scott J, Leboyer M, Hickie I, Berk M, Kapczinski F, Frank E, Kupfer D and McGorry P (2013) Clinical staging in psychiatry: a cross-cutting model of diagnosis with heuristic and practical value. British Journal of Psychiatry 202, 243–245. 10.1192/bjp.bp.112.110858 23549937

[ref45] Scott J, Davenport TA, Parker R, Hermens DF, Lind PA, Medland SE, Wright MJ, Martin NG, Gillespie NA and Hickie IB (2018) Pathways to depression by age 16 years: examining trajectories for self-reported psychological and somatic phenotypes across adolescence. Journal of Affective Disorders 230, 1–6. 29355726 10.1016/j.jad.2017.12.007

[ref46] Simons LE, Elman I and Borsook D (2014) Psychological processing in chronic pain: a neural systems approach. Neuroscience and Biobehavioral Reviews 39, 61–78. 24374383 10.1016/j.neubiorev.2013.12.006 PMC3944001

[ref47] Slater H, Jordan JE, Chua J, Schütze R and Briggs AM (2016) Young People's Experiences of Living with Persistent Pain, Their Interactions with Health Services and Their Needs and Preferences for Pain Management Including Digital Technologies. Melbourne: Arthritis and Osteoporosis Victoria and Arthritis and Osteoporosis Western Australia.

[ref48] Spitzer RL, Kroenke K, Williams JB and Lowe B (2006) A brief measure for assessing generalised anxiety disorder: the GAD-7. Archives of Internal Medicine 166, 1092–1097. 16717171 10.1001/archinte.166.10.1092

[ref49] Swain MS, Henschke N, Kamper SJ, Gobina I, Ottová-Jordan V and Maher CG (2014) An international survey of pain in adolescents. BMC Public Health 14, 447–447. 24885027 10.1186/1471-2458-14-447 PMC4046513

[ref50] Swanson SA, Lindenberg K, Bauer S and Crosby RD (2012) A Monte Carlo investigation of factors influencing latent class analysis: an application to eating disorder research. International Journal of Eating Disorders 45, 677–684. 21882219 10.1002/eat.20958

[ref51] Torrance GW (1987) Utility approach to measuring health-related quality of life. Journal of Chronic Diseases 40, 593–603. 3298297 10.1016/0021-9681(87)90019-1

[ref52] Ulbricht CM, Chrysanthopoulou SA, Levin L and Lapane KL (2018) The use of latent class analysis for identifying subtypes of depression: a systematic review. Psychiatry Research 266, 228–246. 29605104 10.1016/j.psychres.2018.03.003 PMC6345275

[ref53] Ware JE, Kosinski M and Keller SD (1996) A 12 item short form health survey: construction of scales and preliminary tests of reliability and validity. Medical Care 34, 220–233. 8628042 10.1097/00005650-199603000-00003

[ref54] Weitkamp K, Daniels JK, Romer G and Wiegand-Grefe S (2013) Health-related quality of life of children and adolescents with mental disorders. Health and Quality of Life Outcomes 11, 129. 23902824 10.1186/1477-7525-11-129 PMC3733630

[ref55] Whoqol Group (1995) The World Health Organization Quality of Life assessment (WHOQOL): position paper from the World Health Organization. Social Sciences and Medicine 41, 1403–1409. 10.1016/0277-9536(95)00112-k 8560308

[ref56] Zahn-Waxler C, Shirtcliff EA and Marceau K (2008) Disorders of childhood and adolescence: gender and psychopathology. Annual Review of Clinical Psychology 4, 275–303. 10.1146/annurev.clinpsy.3.022806.091358 18370618

