# Supplementary material for: Heterogeneity of quality of life in young people attending primary mental health services
Source: Epidemiol Psychiatr Sci. 2022 Jul 20;31:e55. doi: 10.1017/S2045796022000427 (PMC9305730; doi:10.1017/S2045796022000427)
Supplement: Supplementary file 1 [file S2045796022000427sup001.docx]

Supplementary Materials: Heterogeneity of quality of life in young people attending primary mental health services

# Detailed statistical methods

## Network analysis

Pairwise associations between individual items were first evaluated using the polychoric correlation coefficient (*r_pc_*) based on **polychoric** function from **psych** package (Revelle, 2020). Polychoric correlation was used instead of Pearson’s (*r*) correlation due to the fact that it provides a more stable estimate of latent factors (Holgado-Tello *et al.*, 2010). A multidimensional scaling (MDS) network plot (Jones *et al.*, 2018) was then used to visualise these correlation coefficients to determine whether the individual items within a dimension were measuring the latent construct depicted by the dimension. The MDS network plot was implemented using **qgraph** function from **qgraph** package. As the distance between the nodes(items) on the graph can be directly interpreted as the strength of the association, the network plot can assist to determine whether the individual items within a dimension were measuring the latent construct depicted by the dimension.

## Latent class analysis

LCA was used to empirically evaluate the possible heterogeneous groups. LCA is a clustering algorithm, in particular, a mixture model (also known as hidden or latent Markov models), where the latent construct is assumed to be categorical (commonly referred to as latent classes or clusters), and there are no prior hypotheses about the nature of the class categorisations (Hagenaars and McCutcheon, 2002; McLachlan and Peel, 2004). LCA has been widely used in psychology and psychiatry (Petersen *et al.*, 2019; Ulbricht *et al.*, 2018) as multidimensional heterogeneity in the presentation and course of mental disorders is difficult to capture with traditional statistical methods such as regression models and cut-off threshold evaluations (MacCallum *et al.*, 2002).

In this study, LCA was chosen over other types of clustering algorithms, such as k-means and hierarchical clustering, due to its ability to directly modelling the distribution of individual responses without the need of estimating distances between observations (problematic when dimensionality increases) and better performance with overlapping clusters (Steinley and Brusco, 2011). LCA was fitted using the **depmixS4** package in **R** (Visser and Speekenbrink, 2010; McCutcheon, 1987). Individual items were assumed to be multinomially distributed due to skewness and limited response categories for each item.

### Cross Validation (CV) for selecting the number of classes

In order to improve model stability, 10-fold cross-validation (CV) and leave-one-site-out (LOSO) CV were used to identify the best number of classes (also known as clusters) instead of the traditional log-likelihood ratio test (Grimm *et al.*, 2017; Payne *et al.*, 2011). Ten-fold CV is a widely applied method in machine learning for parameter selection, which randomly segments the dataset into 10 groups and carries out modelling on each combination of 9 groups (known as 9-fold training data) and evaluation on the remaining groups (known as testing data). This method reduces the likelihood of overfitting based on observed data (Grimm *et al.*, 2017). Compared with other resampling methods, K-folds CV explores all possible combinations of small data sections without high computational cost. Therefore, it was chosen in this study.

The data were collected from 5 *headspace* centres, which allows us to evaluate modelling stability across data collection sites using LOSO CV. LOSO CV is a similar method of validating the robustness of modelling results with each training dataset being all data except for data collected from one site, which will act as the testing dataset (Payne *et al.*, 2011).

In this study, we use training datasets to identify the best number of classes and testing datasets to evaluate the consistency of fitting indexes across different smaller samples. The Bayesian Information Criterion (BIC) was used as the main fitting index for choosing class numbers because it is easy to compute, more accurate and less sensitive to sample size (Nylund *et al.*, 2007). BIC was used rather than sample adjusted BIC, as there is no clear evidence that adjusted BIC perform better than BIC (Nylund *et al.*, 2007). Akaike Information Criteria (AIC) and log-likelihood were also used to guide interpretation. The model with lower BIC and AIC values and higher log-likelihood was considered a better fit to the data. Individuals were assigned to the best fitting latent class model using the estimated posterior class probabilities. Rand index was used to measure agreements in overlapping samples between each pair of folds (Rand, 1971).

In supervised learning algorithms, prediction error from the model established using the training data can be directly evaluated with the testing data (out of sample prediction). However, as the latent subgroup is unknown, the modelling error cannot be directly evaluated using testing data in clustering algorithms. Although a few CV methods for clustering have been proposed (Fu and Perry, 2020; Tarekegn *et al.*, 2020), there are often limited uses for these methods and there is no consensus on the appropriate approach across different clustering algorithms. As the fitted LCA model involves with many modelling parameters which could be problematic in small samples in Ten-fold CV and LOSO CV. Therefore, the testing data in was not used in selecting number of classes. To avoid possible chance finding, we also conducted split-half CV and evaluated whether similar clusters can be identified in the two random subsamples (results not shown).

### Sensitivity analysis

As there were moderate level of correlation between individual items of AQoL-6D, the conditional independence assumption can be violated, which can potentially bias model parameters and posterior classifications (Albert and Dodd, 2004). However, diagnosing violation of conditional independence can be difficult, and best practice of modelling conditional dependence is less clear. Therefore, we used K-means clustering to validate LCA results. To achieve this, we first conducted the principal comment analysis (assuming individual variables are continuous). Then, the first five principal comments were used to conduct a k-means clustering (using Euclidean distance). The k-means clustering results were then compared with LCA.

## Profiles of latent classes

The meaningfulness of the latent classes identified was first evaluated with respect to distributions of **standardised dimension** and **total utility scores.** The latent classes were labelled according to the level of impairments in QoL across dimensions.

We also validated the classes against demographic, social and clinical factors. The descriptive statistics (proportions for binary and categorical variables and median and interquartile range [IQR] for continuous variables) were used to broadly compare between class differences in demographic, social and clinical factors. Multivariate multinomial logistic regression models using the **multinom** function from **nnet** package (Venables and Ripley, 2013) were then applied to class membership to broadly understand whether external demographic, clinical and functioning factors were associated with the identified group membership.

Due to possible overlap between clinical diagnosis/severity and self-reported symptoms, two models were fitted. In the first model, only demographics (age, sex at birth and LGBTIQ), social factors (region of residency and NEET status), primary diagnosis and clinical staging were included. Self-reported clinical symptoms measured by PHQ9, GAD7, SIQ-Jr and PSQI were added in the second model. A small proportion of data were missing across different risk factors, therefore missingness was addressed using multiple imputation using chained equations (MICE) using **mice** function from **mice** package (van Buuren and Groothuis-Oudshoorn, 2011). All regression models were run on 5 imputed datasets and the results were combined using Rubin’s rules (Toutenburg, 1990).

**References**

**Albert PS and Dodd LE**. (2004) A cautionary note on the robustness of latent class models for estimating diagnostic error without a gold standard. *Biometrics* **60**: 427-435. <https://doi.org/10.1111/j.0006-341X.2004.00187.x>

**Fu W and Perry PO**. (2020) Estimating the Number of Clusters Using Cross-Validation. *Journal of Computational and Graphical Statistics* **29**: 162-173. <https://doi.org/10.1080/10618600.2019.1647846>

**Grimm KJ, Mazza GL and Davoudzadeh P**. (2017) Model selection in finite mixture models: A k-fold cross-validation approach. *Structural Equation Modeling* **24**: 246-256

**Hagenaars JA and McCutcheon AL**. (2002) *Applied latent class analysis,* Cambridge: Cambridge University Press.

**Holgado-Tello FP, Chancon-Moscoso S, Barbero-Garcia I and Villa-Abad E**. (2010) Polychoric versus Pearson correlations in exploratory and confirmatory factor analysis of ordinal variables. *Quality and Quantity* **44**: 153-166

**Jones PJ, Mair P and McNally RJ**. (2018) Visualizing psychological networks: A tutorial in R. *Frontiers in Psychology* **9**: 1742

**MacCallum R, Zhang S, Preacher K and Rucker D**. (2002) On the Practice of Dichotomizing Quantitative Variables. *Psychological Methods* **7**: 19-40. <https://doi.org/10.1037/1082-989X.7.1.19>

**McCutcheon AL**. (1987) *Latent class analysis*: Sage.

**McLachlan G and Peel D**. (2004) *Finite mixture models,* New York: John Wiley and Sons.

**Nylund KL, Asparouhov T and Muthén BO**. (2007) Deciding on the Number of Classes in Latent Class Analysis and Growth Mixture Modeling: A Monte Carlo Simulation Study. *Structural Equation Modeling: A Multidisciplinary Journal* **14**: 535-569. <https://doi.org/10.1080/10705510701575396>

**Payne RJ, Telford RJ, Blackford JJ, Blundell A, Booth RK, Charman DJ, Lamentowicz Ł, Lamentowicz M, Mitchell EAD, Potts G, Swindles GT, Warner BG and Woodland W**. (2011) Testing peatland testate amoeba transfer functions: Appropriate methods for clustered training-sets. *The Holocene* **22**: 819-825. <https://doi.org/10.1177/0959683611430412>

**Petersen KJ, Qualter P and Humphrey N**. (2019) The Application of Latent Class Analysis for Investigating Population Child Mental Health: A Systematic Review. *Frontiers in Psychology* **10**: 1214. <https://doi.org/10.3389/fpsyg.2019.01214>

**Revelle W**. (2020) psych: Procedures for Personality and Psychological Research. 2.0.12 ed. Illinois Northwestern University.

**Steinley D and Brusco MJ**. (2011) Evaluating mixture modeling for clustering: Recommendations and cautions. *Psychological Methods* **16**: 63-79. <https://doi.org/10.1037/a0022673>

**Tarekegn AN, Michalak K and Giacobini M**. (2020) Cross-Validation Approach to Evaluate Clustering Algorithms: An Experimental Study Using Multi-Label Datasets. *SN Computer Science* **1**: 263. <https://doi.org/10.1007/s42979-020-00283-z>

**Toutenburg H**. (1990) Rubin, D.B.: Multiple imputation for nonresponse in surveys. *Statistical Papers* **31**: 180-180. <https://doi.org/10.1007/BF02924688>

**Ulbricht CM, Chrysanthopoulou SA, Levin L and Lapane KL**. (2018) The use of latent class analysis for identifying subtypes of depression: A systematic review. *Psychiatry Research* **266**: 228-246. <https://doi.org/10.1016/j.psychres.2018.03.003>

**van Buuren S and Groothuis-Oudshoorn K**. (2011) mice: Multivariate Imputation by Chained Equations in R. *Journal of Statistical Software* **45**: 1 - 67. <https://doi.org/10.18637/jss.v045.i03>

**Venables WN and Ripley BD**. (2013) *Modern applied statistics with S-PLUS*: Springer Science & Business Media.

**Visser I and Speekenbrink M**. (2010) depmixS4: An R Package for Hidden Markov Models. *Journal of Statistical Software; Vol 1, Issue 7 (2010)*

**Supplementary Tables and Figures.**

**Figure A1**

Distribution of Individual AQoL-6D Items in the 1067 Young People Presenting to Five headspaces Services


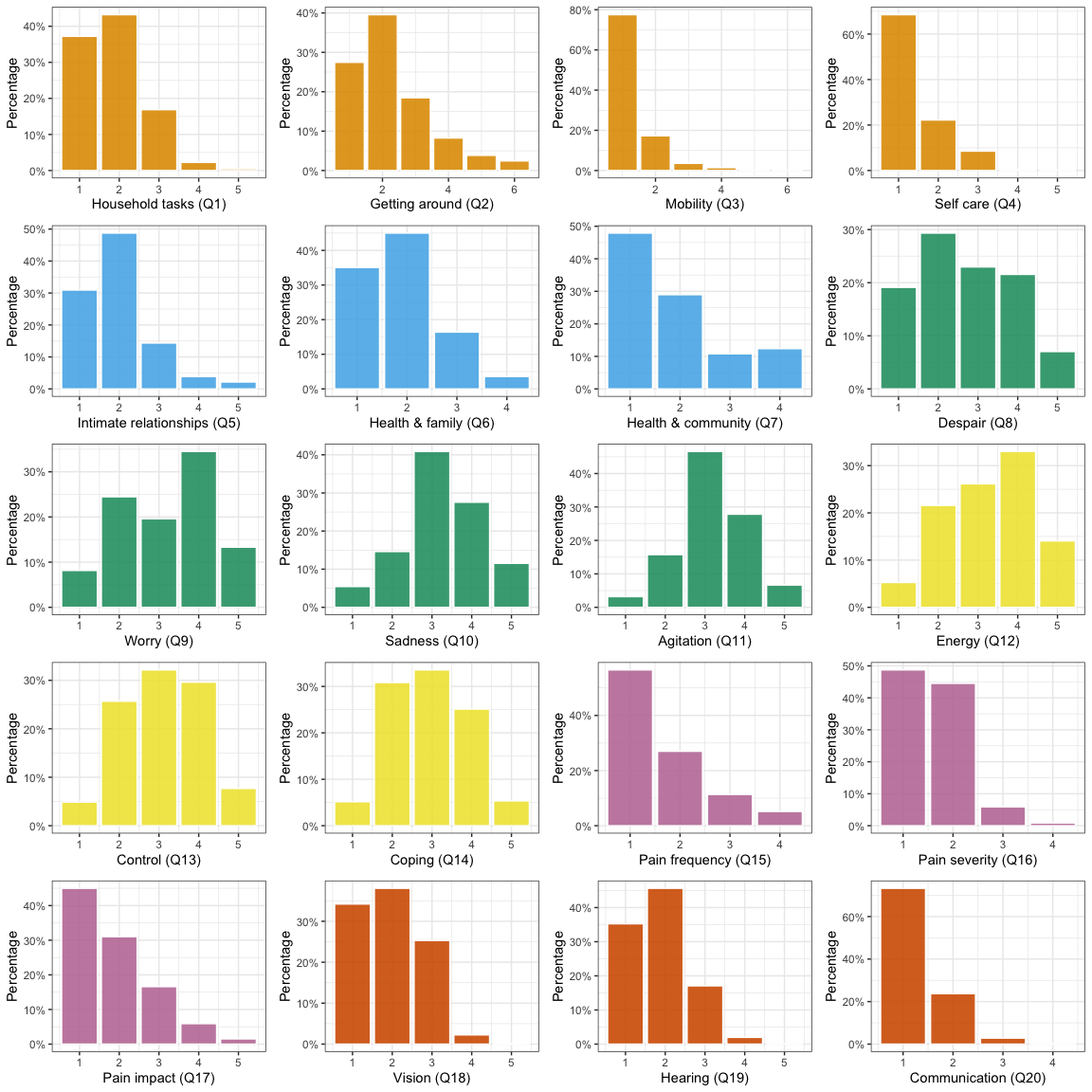


Note: Dimensions of AQoL-6D include: Independent living (orange), Relationships (blue), Mental health (green), Coping(yellow), Pain (pink) and Sense (orange)

**Figure A2**

Standardised Distributions of the AQoL-6D Dimensions with Scores Ranging from 0 “Worst Qol” to 100 “Best Qol”.


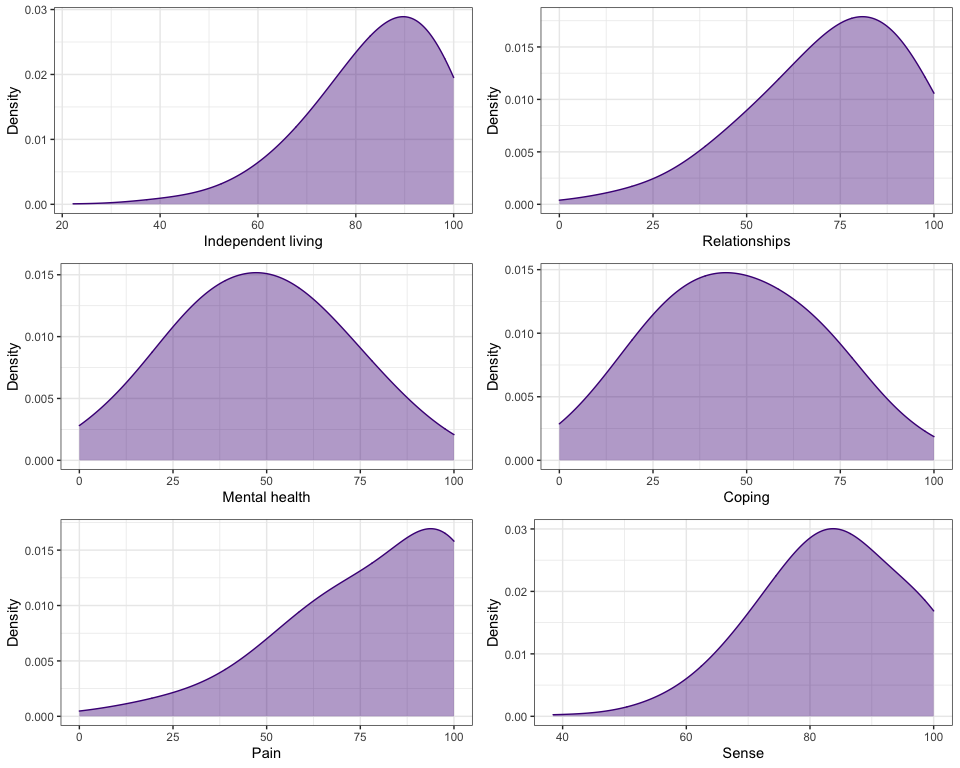


**Figure A3**

Pairwise Polychoric (r_pc_) Correlation Matrix of the 20 AQoL-6D Items


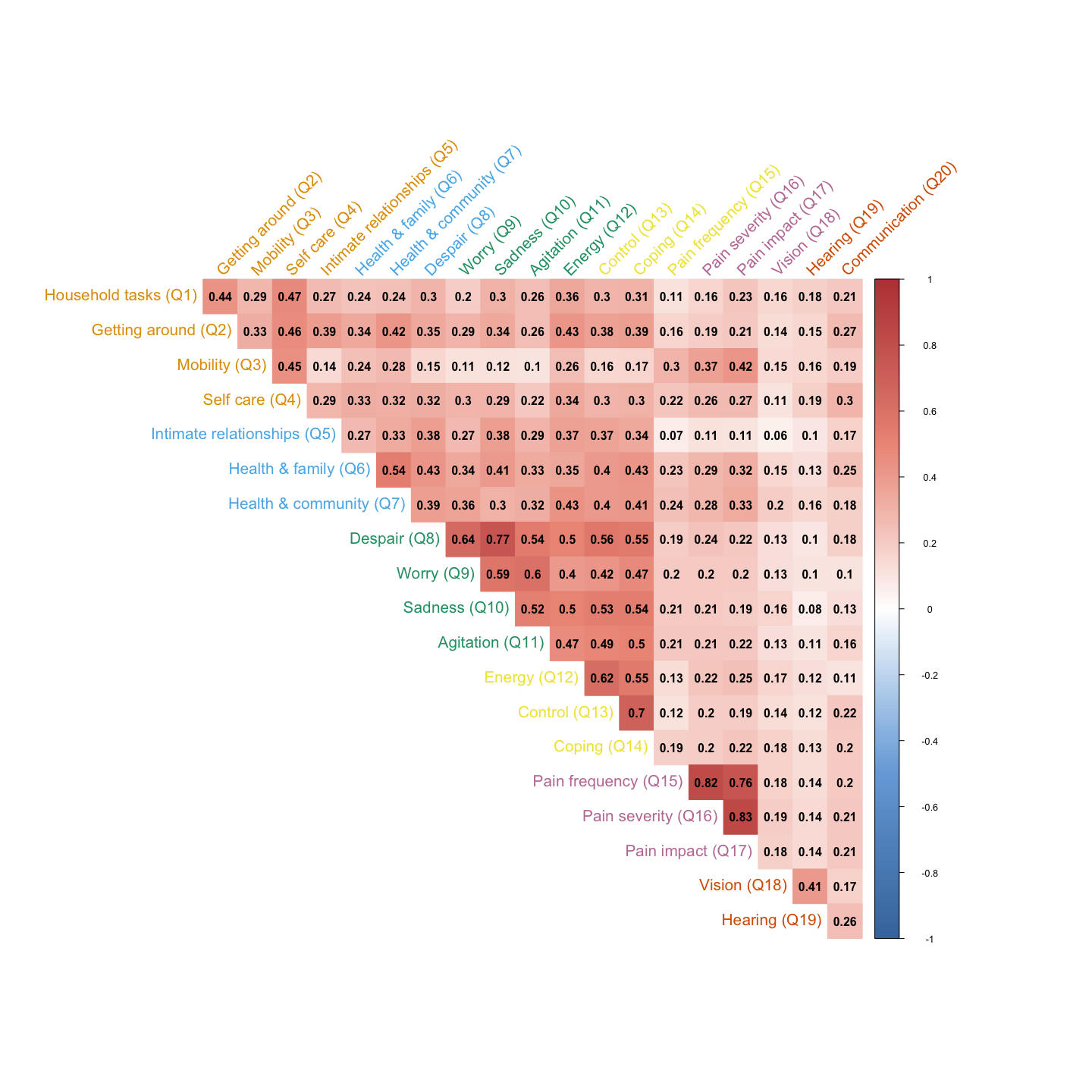


Note: Dimensions of AQoL-6D include: Independent living (orange), Relationships (blue), Mental health (green), Coping(yellow), Pain (pink) and Sense (orange)

**Figure A4**

Model Fitting Indexes from Individual Models of 10-Fold CV training datasets


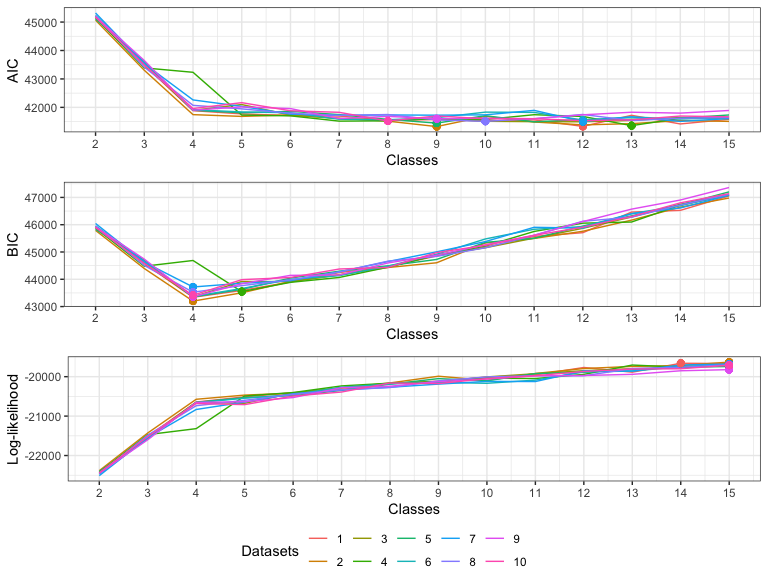


Note: AIC: Akaike Information Criteria; BIC; Bayesian Information Criteria; dots indicate the lowest (AIC and BIC) or highest fitting index (log-likelihood) for the individual dataset. Lower AIC and BIC, and higher Log-likelihood indicate better model fit. Log-likelihood increases with the complexity of the model, whereas AIC and BIC penalise the number of model parameters based on Log-likelihood. Compared with AIC, BIC implements a stronger level of penalisation with a more complex model. In this case, the 4-class solution was supported consistently across most of the training datasets using BIC, whereas both AIC and Log-likelihood reaches an elbow point at the four classes solution.

**Figure A5**

Model Fitting Indexes from Individual Models of Leave-Centre-Out CV training datasets


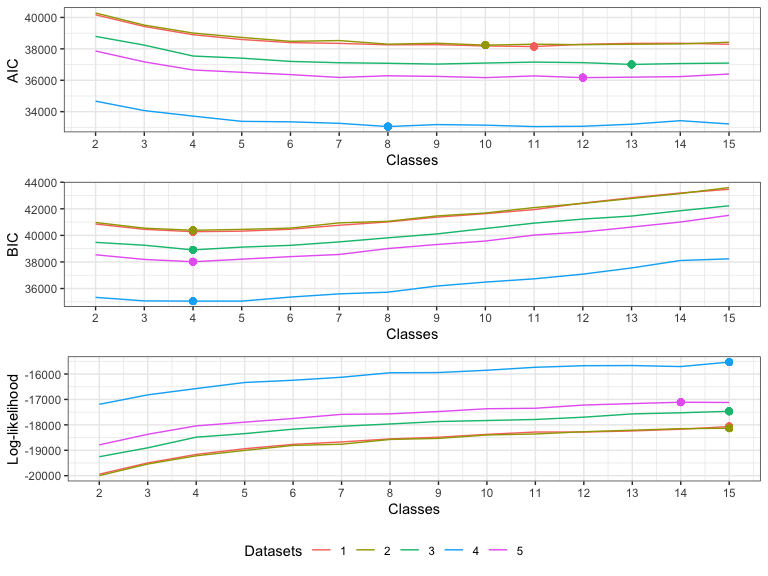


Note: AIC, Akaike Information Criteria; BIC; Bayesian Information Criteria; dots indicate the lowest (AIC and BIC) or highest fitting index (log-likelihood) for the individual dataset. Lower AIC and BIC, and higher Log-likelihood indicate better model fit. Log-likelihood increases with the complexity of the model, whereas AIC and BIC penalise the number of model parameters based on Log-likelihood. Compared with AIC, BIC implements a stronger level of penalisation with a more complex model. In this case, the 4-class solution was supported consistently across all training datasets using BIC.

**Table A1**

Means (±standard deviations) and median (Q_1_, Q_3_) for the overall cohort and separately for the four predicted classes on AQoL-6D standardized dimension and utility scores

|  | Overall,  N = 1,067 | No/Mild,  n = 280 | Moderate-Phy,  n = 296 | Moderate-Psy,  n = 304 | Severe,  n = 187 |
| --- | --- | --- | --- | --- | --- |
| **AQoL-6D standardised dimension scores (range 0-100)** | | | | | |
| **Independent living** |  |  |  |  |  |
| Mean (SD) | 84 (13) | 92 (9) | 85 (10) | 85 (10) | 70 (15) |
| Median (Q1, Q3) | 89 (78, 94) | 94 (89, 100) | 83 (78, 94) | 89 (78, 94) | 72 (61, 78) |
| **Relationships** |  |  |  |  |  |
| Mean (SD) | 73 (20) | 89 (11) | 74 (14) | 71 (17) | 49 (21) |
| Median (Q1, Q3) | 80 (60, 90) | 90 (80, 100) | 80 (70, 80) | 70 (60, 80) | 50 (40, 60) |
| **Mental health** |  |  |  |  |  |
| Mean (SD) | 48 (22) | 72 (14) | 51 (13) | 40 (14) | 21 (13) |
| Median (Q1, Q3) | 50 (31, 62) | 75 (62, 81) | 50 (44, 56) | 38 (31, 50) | 25 (12, 31) |
| **Coping** |  |  |  |  |  |
| Mean (SD) | 47 (22) | 71 (15) | 49 (15) | 39 (15) | 23 (13) |
| Median (Q1, Q3) | 50 (33, 67) | 75 (65, 75) | 50 (42, 58) | 42 (25, 50) | 25 (17, 33) |
| **Pain** |  |  |  |  |  |
| Mean (SD) | 79 (22) | 91 (14) | 63 (14) | 96 (8) | 57 (23) |
| Median (Q1, Q3) | 80 (65, 100) | 100 (80, 100) | 70 (50, 70) | 100 (90, 100) | 60 (40, 70) |
| **Sense** |  |  |  |  |  |
| Mean (SD) | 84 (12) | 89 (9) | 82 (12) | 85 (11) | 77 (12) |
| Median (Q1, Q3) | 85 (77, 92) | 92 (85, 100) | 85 (77, 92) | 85 (77, 92) | 77 (69, 85) |
| **AQoL-6D utility score (range 0-1)** | | | | | |
| Mean (SD) | 0.59 (0.24) | 0.87 (0.10) | 0.54 (0.14) | 0.59 (0.12) | 0.25 (0.10) |
| Median (IQR) | 0.60 (0.43, 0.77) | 0.87 (0.82, 0.94) | 0.55 (0.45, 0.64) | 0.60 (0.50, 0.68) | 0.23 (0.16, 0.30) |
